# A multi-omics landscape of programmed cell death in acetaminophen-induced acute kidney injury

**DOI:** 10.1080/0886022X.2025.2580064

**Published:** 2025-11-17

**Authors:** Jianxin Zheng, Peng Lai, Jiaheng Wu, Yuqiu Li, Fengxian Chen, Dong Zhu

**Affiliations:** a Department of Urology, Zhongshan Hospital (Xiamen), Fudan University, Xiamen, China; b Department of Kidney Transplantation, Zhongshan Hospital, Fudan University, Shanghai, China; c Shanghai Key Laboratory of Organ Transplantation, Shanghai, China; d Department of Transfusion, Zhongshan Hospital (Xiamen), Fudan University, Xiamen, China; e Department of Neurology, The Second Affiliated Hospital of Xiamen Medical College, Xiamen, China

**Keywords:** Drug-induced kidney injury, toxic nephropathy, cell death modalities, multi-omics AKI, kinase inhibition therapy

## Abstract

Acetaminophen (APAP) overdose is a known cause of acute kidney injury, yet the underlying molecular mechanisms remain incompletely understood. In this study, we conducted integrated transcriptomic, proteomic, and phosphoproteomic analyses of kidney tissues from mice with early-stage APAP-induced nephrotoxicity and corresponding controls. A total of 884 genes related to 13 distinct forms of programmed cell death (PCD)—including alkaliptosis, apoptosis, autophagy, cuproptosis, disulfidptosis, entotic cell death, ferroptosis, lysosome-dependent cell death, necroptosis, netotic cell death, oxeiptosis, parthanatos, and pyroptosis—were systematically evaluated. Gene set variation analysis was employed to assess the activity of these pathways in APAP-injured kidneys. Moreover, phosphokinase profiling and *in vivo* inhibition of protein kinase B (AKT) and extracellular signal–regulated kinase were performed to identify potential therapeutic strategies. Pathway enrichment across transcriptomic and proteomic datasets consistently pointed to drug metabolism—particularly cytochrome P450—as a central player. Transcriptomic profiling highlighted six PCD pathways—alkaliptosis, cuproptosis, disulfidptosis, lysosome-dependent cell death, netotic cell death, and pyroptosis—as notably activated in response to APAP exposure. Proteomic analysis further revealed enrichment of eight PCD pathways, including alkaliptosis, apoptosis, entotic cell death, ferroptosis, necroptosis, netotic cell death, oxeiptosis, and pyroptosis. Therapeutically, *in vivo* inhibition of AKT significantly alleviated renal injury, as demonstrated by improved histopathology, reduced neutrophil gelatinase-associated lipocalin and blood urea nitrogen and suppression of ferroptosis mediators TFRC and ACSL4. Concurrently, it enhanced phosphorylation of p70S6K and FOXO, reflecting improved survival signaling and reduced apoptosis. Together, these findings demonstrate that multiple PCD pathways contribute to early APAP-induced nephrotoxicity and nominate AKT as a central regulatory hub, which merits further exploration in translational nephrotoxicity research.

## GRAPHICAL ABSTRACT

**Figure:**
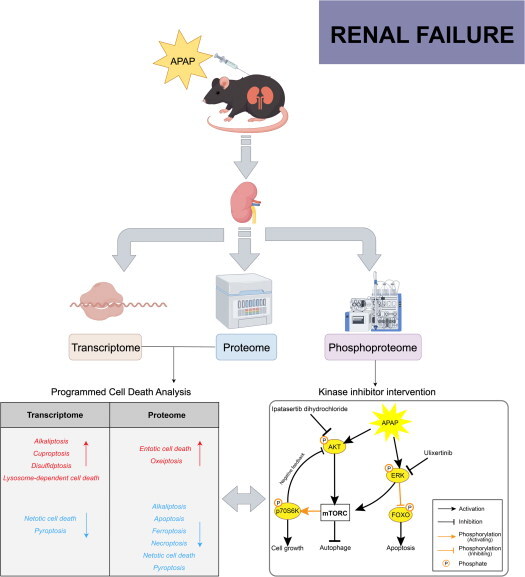

## 1. Introduction

Acetaminophen (APAP), also known as paracetamol, is a widely used antipyretic and analgesic agent, and has been recommended as a first-line treatment for symptomatic relief in coronavirus disease 2019 (COVID-19) [1–4]. When used at therapeutic doses (≤ 4 g/day for adults; ≤ 60 mg/kg/day for children), APAP is generally safe. However, overdose (≥ 7.5 g/day for adults; ≥ 100 mg/kg/day for children) can lead to severe liver and kidney toxicity, potentially resulting in organ failure [5–8]. Hepatotoxicity is the most well-characterized consequence of APAP overdose and is primarily mediated by the cytochrome P450–dependent formation of the reactive metabolite N-acetyl-p-benzoquinone imine (NAPQI). NAPQI depletes intracellular glutathione (GSH), promotes oxidative stress, and causes mitochondrial damage in hepatocytes [9]. While hepatic injury remains the predominant concern, renal damage is reported in approximately 2%–10% of APAP overdose cases, and 1%–2% of patients may develop acute renal failure [10–13]. Notably, nephrotoxicity can occur independently of hepatotoxicity, highlighting the importance of understanding extrahepatic effects of APAP [14–16].

Although the liver is the primary target of APAP toxicity, the kidneys are also vulnerable due to their role in drug clearance. In the kidney, cytochrome P450 2E1 (CYP2E1) expressed in proximal tubular cells catalyzes the formation of NAPQI, contributing to local GSH depletion and covalent binding of NAPQI to proteins, initiating cellular injury [17,18]. However, the subsequent molecular events following APAP metabolism in the kidney remain controversial. For example, Hua et al. reported that the mitochondrial complex I inhibitor rotenone alleviated APAP-induced renal injury by suppressing oxidative stress and inflammation [19]. Shen et al. demonstrated that IL-22 improved renal outcomes by attenuating mitochondrial dysfunction and NLRP3 inflammasome activation [20]. In contrast, Lorz et al. further investigated the molecular mechanisms of APAP-induced nephrotoxicity and reported that the mitochondrial apoptosis pathway was not involved, as there was no evidence of mitochondrial membrane potential loss, BAX translocation, or cytochrome c release in vitro [21]. Consistently, Akakpo et al. demonstrated that while APAP-protein adducts can be observed throughout kidney homogenates, mitochondrial fractions do not exhibit such adduct formation [22], and found that CYP2E1 in the kidney is primarily localized in the endoplasmic reticulum (ER) rather than mitochondria, and that ER-localized metabolism leads to sustained ER stress, activation of procaspase-12, and apoptosis [23]. These findings suggest that mechanisms of APAP-induced nephrotoxicity may differ substantially from hepatic injury and are likely organ-specific.

Programmed cell death (PCD) plays a critical role in maintaining tissue homeostasis and eliminating damaged cells. Since the term ‘apoptosis’ was introduced by Kerr et al. in 1972, multiple non-apoptotic forms of PCD have been identified, including alkaliptosis, apoptosis, autophagy, cuproptosis, disulfidptosis, entotic cell death, ferroptosis, lysosome-dependent cell death, necroptosis, netotic cell death, oxeiptosis, parthanatos, and pyroptosis. These pathways are increasingly recognized as key contributors to acute kidney injury (AKI) and are frequently targeted by renoprotective therapies. For instance, APAP has been shown to induce ER stress–mediated apoptosis *via* CYP2E1 activity in renal proximal tubules, a process that can be inhibited by 4-methylpyrazole, but not by N-acetylcysteine, indicating a mechanism distinct from hepatotoxicity [23]. Moreover, apoptosis, necroptosis, and pyroptosis have been implicated in AKI pathogenesis, often acting in a compensatory or synergistic manner [24].

In this study, we performed integrated transcriptomic, proteomic, and phosphoproteomic analyses in a mouse model of early-stage APAP-induced kidney injury. Our goal was to characterize molecular alterations and identify key signaling pathways associated with nephrotoxicity, with a particular focus on PCD mechanisms and phosphokinase activity. Given the clinical importance of APAP-induced renal injury, uncovering novel therapeutic targets is of high relevance. Importantly, kidney-specific mechanisms of APAP toxicity differ from hepatic pathways and require distinct therapeutic approaches, underscoring the need to study renal injury as an independent entity. In line with KDIGO guidelines [25], we evaluated kidney function primarily using serum creatinine (Cr), complemented by blood urea nitrogen (BUN) and neutrophil gelatinase-associated lipocalin (NGAL) as translational biomarkers [26–28]. To this end, we selected candidate kinases based on phosphoproteomic profiling and evaluated their roles through *in vivo* inhibition studies, leveraging the central importance of protein phosphorylation in cellular signaling and injury response.

## 2. Materials and methods

### 2.1. Animals and establishment of APAP-induced kidney injury model

Ten-week-old male C57BL/6 mice were obtained from the Shanghai Model Organisms Center, Inc. (Shanghai, China), and housed under specific pathogen-free (SPF) conditions. Mice were fasted overnight for 16 h prior to APAP treatment. To induce acute kidney injury, mice (*n* = 4) received an intraperitoneal injection of APAP overdose (300 mg/kg body weight) and were sacrificed 6 h post-injection. Control mice received an equivalent volume of PBS (15 mL/kg) *via* intraperitoneal injection (*n* = 4). APAP was purchased from Sigma-Aldrich (St. Louis, MO) and dissolved in pre-warmed PBS (20 mg/ml). All animal experiments were performed in accordance with the guidelines for the care and use of laboratory animals and were approved by the Institutional Animal Care and Use Committee of Zhongshan Hospital, Fudan University and the Animal Ethics Committee of Shanghai Model Organisms Center (2024-0081).

### 2.2. RNA-Seq analysis

Kidney tissues were collected from APAP-induced kidney injury mice (*n* = 4) and PBS-injected controls (*n* = 4). Total RNA was extracted using TRIzol reagent following the manufacturer’s instructions. RNA purification, reverse transcription, library preparation, and sequencing were conducted at Shanghai Majorbio Bio-Pharm Biotechnology Co., Ltd. (Shanghai, China) using the Illumina NovaSeq 6000 platform (Illumina, San Diego, CA, USA). Transcript expression levels were calculated using the transcripts per kilobase per million mapped reads (TPM) method. Genes exhibiting at least a 2-fold change in expression and a *P_adjust_* value less than 0.05 were considered differentially expressed genes (DEGs). The data discussed in this publication have been deposited in NCBI’s Gene Expression Omnibus [29] and are accessible through GEO Series accession number GSE295115.

### 2.3. Real-Time quantitative PCR (RT-qPCR) validation

RT-qPCR was performed using the PowerUp SYBR Green Master Mix Kit (Applied Biosystems, A25741). The cycling protocol consisted of an initial step at 50 °C for 2 min and 95 °C for 2 min, followed by 40 cycles of 95 °C for 15 s and 60 °C for 30 s. Final steps included incubations at 95 °C for 15 s, 60 °C for 1 min, and 95 °C for 45 s. The top 10 DEGs (*P_adjust_* value < 0.05, TPM > 50) were selected for validation (Supplementary Fig. 1). Primer sequences were synthesized by Sangon Biotech (Shanghai, China) and are listed in Table 1. Relative gene expression was calculated using the 2^−ΔΔCt^ method, with *Gapdh* serving as the endogenous control.

**Table 1.** Lists of primers sequences in RT-qPCR.

Gene Name	Forward (5’ – 3’)	Reverse (5’ – 3’)
*Cbr1*	*TCAATGACGACACCCCCTTC*	*CCTCTGTGATGGTCTCGCTTC*
*Inmt*	*GCAGAGCAGGAAATCGTAAAGT*	*GGGGTGTAGTCAGTGACAATGAT*
*Plin2*	*GACCTTGTGTCCTCCGCTTAT*	*CAACCGCAATTTGTGGCTC*
*Sqstm1*	*AGGATGGGGACTTGGTTGC*	*TCACAGATCACATTGGGGTGC*
*Acsm2*	*TGGGGGAATGAGATTTCCTGC*	*CTTCACTCAGTTCTCGGAAGC*
*Txnrd1*	*CCCACTTGCCCCAACTGTT*	*GGGAGTGTCTTGGAGGGAC*
*Mt2*	*GCCTGCAAATGCAAACAATGC*	*AGCTGCACTTGTCGGAAGC*
*Mt1*	*AAGAGTGAGTTGGGACACCTT*	*CGAGACAATACAATGGCCTCC*
*Ces2c*	*GCCAACCCCATCAGAAACACA*	*TTCAGCATGTCAAGATTTTGCAG*
*Cndp2*	*GACCGCTACGTCAAGAAACTT*	*CCCGTAAATGCACACGGTT*
*Gapdh*	*AGGTCGGTGTGAACGGATTTG*	*TGTAGACCATGTAGTTGAGGTCA*

### 2.4. Programmed cell death (PCD)-related gene sets and pathway activity analysis

To investigate the activity of programmed cell death (PCD) pathways in APAP-induced kidney injury, we curated representative gene sets for 13 distinct PCD modalities from multiple publicly accessible sources, including KEGG, GeneCards, MSigDB, Reactome, and peer-reviewed literature [30–34]. The finalized gene list for each PCD subtype is detailed in Supplementary Table 1. Specifically, these pathways included alkaliptosis (7 genes), apoptosis (136 genes), autophagy (151 genes), cuproptosis (14 genes), disulfidptosis (4 genes), entotic cell death (15 genes), ferroptosis (64 genes), lysosome-dependent cell death (255 genes), necroptosis (159 genes), netotic cell death (17 genes), oxeiptosis (26 genes), parthanatos (9 genes), and pyroptosis (27 genes), comprising a total of 884 unique PCD-related genes. To quantify pathway-level activity changes in transcriptomic and proteomic datasets, we utilized Gene Set Variation Analysis (GSVA). GSVA is a nonparametric, unsupervised method that estimates gene set enrichment scores on a per-sample basis, allowing assessment of pathway dynamics across conditions without requiring predefined phenotypic groupings. The GSVA algorithm was implemented in R using the ‘GSVA’ package, and enrichment scores were calculated for all 13 PCD pathways. These scores enabled comparative analyses of pathway activity patterns between control and APAP-treated groups at both transcriptome and proteome levels. R script used for GSVA analysis is detailed in Supplementary materials.

### 2.5. Proteome and phosphoproteome analysis

Kidney tissues from APAP-induced kidney injury mice (*n* = 4) and PBS-injected controls (*n* = 4) were prepared for proteomic and phosphoproteomic analysis. Protein extraction, trypsin digestion, affinity enrichment (IMAC), and LC-MS/MS were carried out by PTM Biolabs Co., Ltd. (Hangzhou, China), following established protocols [35]. The mass spectrometry proteomics and phosphoproteomics data have been deposited to the ProteomeXchange Consortium *via* the PRIDE[36] partner repository with the dataset identifiers PXD063122 and PXD063126.

### 2.6. Kinase prediction and activity score

Kinase-substrate predictions were made using iGPS1.0 software, which utilizes short linear motifs (SLMs) around phosphorylation sites to predict site-specific kinase-substrate relationships (ssKSRs) [37]. Kinase-substrate interactions were annotated using PhosphoSitePlus, a comprehensive resource of PTM sites. The KSEA method was used to predict kinase activity by calculating the mean log2 fold change of kinase substrates. Normalized enrichment scores (z.score) derived from the KSEA algorithm reflect predicted kinase activity. Positive (activated) kinase activity was defined as z.score > 0, and negative (inhibited) kinase activity as z.score < 0.

### 2.7. Kinase-substrate regulatory network construction

Kinase-substrate regulatory networks were constructed using kinases with predicted activity (either positive or negative) and differentially expressed phosphorylation sites (*p* value < 0.05, fold change > 1.5 or < 1/1.5).

### 2.8. Kinase inhibitor dissolution and *In vivo* inhibition experiment

Ipatasertib dihydrochloride (protein kinase B [AKT] inhibitor, HY-15186A), and ulixertinib (extracellular signal–regulated kinase [ERK] inhibitor, HY-15816) were purchased from MedChemExpress (Monmouth Junction, NJ). Inhibitors were dissolved in 5% DMSO + 30% PEG300 + 5% Tween 80 + 60% ddH2O. Male C57BL/6 mice were divided into four groups: control, vehicle, iAKT (ipatasertib dihydrochloride, 100 mg/kg), and iERK (ulixertinib, 100 mg/kg). Inhibitors were administered *via* oral gavage 1 h before APAP injection. The vehicle group received the equal solvent orally. All groups, except the control group, received an intraperitoneal injection of APAP (300 mg/kg). Mice were euthanized 6 h after APAP administration.

### 2.9. Western blot

Samples were lysed in RIPA lysis buffer (Beyotime) and supplemented with a protease and phosphatase inhibitor cocktail (Thermo Scientific, Waltham, MA). Antibodies used include phospho-p70S6 Kinase^Thr389^ (1:1000; 9234, CST), phospho-FoxO1^Thr24^/FoxO3a^Thr32^ (1:1000; 9464, CST), phospho-Akt^Ser473^ (1:1000; 4060, CST), phospho-Erk1/2^Thr202/Tyr204^ (1:1000; 7155, PTM Biolab), LC3B (1:1000; 6384, PTM Biolab), TFRC (1:1000; ab214039, abcam), STEAP3 (1:1000; PA5-102321, Invitrogen), ACSL4 (1:1000; 38493, CST), Bcl-xL (1:1000; ab32370, abcam), BAX (1:1000; ab32503, abcam), β-Actin (1:1000; 4970, CST), GAPDH (1:1000; 2118, CST). The band was detected with ECL Western Blotting Substrate (Beyotime) by a GE Amersham Imager 600 (GE Healthcare).

### 2.10. Histopathological analysis

Kidney tissues were fixed in 4% formaldehyde, embedded in paraffin, sectioned, and stained with hematoxylin-eosin (HE) and periodic acid-Schiff (PAS) for histopathological evaluation. Histological damage was assessed using a blinded scoring system to quantify the extent of renal injury. The severity was graded on a scale from 0 to 4: 0 indicating normal tissue; 1 representing mild damage involving less than 25% of the cortex; 2 indicating moderate damage affecting 25–50% of the cortex; 3 denoting severe damage with 50–75% cortical involvement; and 4 corresponding to extensive damage affecting more than 75% of the cortex [19].

### 2.11. Renal function assessment and enzyme-linked immunosorbent assay (ELISA)

Blood samples were collected and centrifuged at 1200 g for 15 min at room temperature. Serum creatinine (Scr) and blood urea nitrogen (BUN) levels were measured using an automatic analyzer (Hitachi 3500, Tokyo, Japan). Serum neutrophil gelatinase-associated lipocalin (NGAL) was quantified using a commercial ELISA kit (Cat#MLCN20, R&D Systems, Minneapolis, MN).

### 2.12. Statistical analysis

Statistical analyses were performed using GraphPad Prism version 9.0 (GraphPad Software, San Diego, CA, USA). Comparisons among multiple groups were conducted using one-way analysis of variance (ANOVA), followed by Tukey’s *post hoc* test. For comparisons between two groups, an unpaired Student’s t-test was used. Data are presented as mean ± standard deviation (SD) for normally distributed variables. A *p*-value < 0.05 was considered statistically significant.

## 3. Results

### 3.1. Global transcriptomic, proteomic, and phosphoproteomic profiling of the kidney in control and APAP-induced kidney injury mice

**Figure 1.**
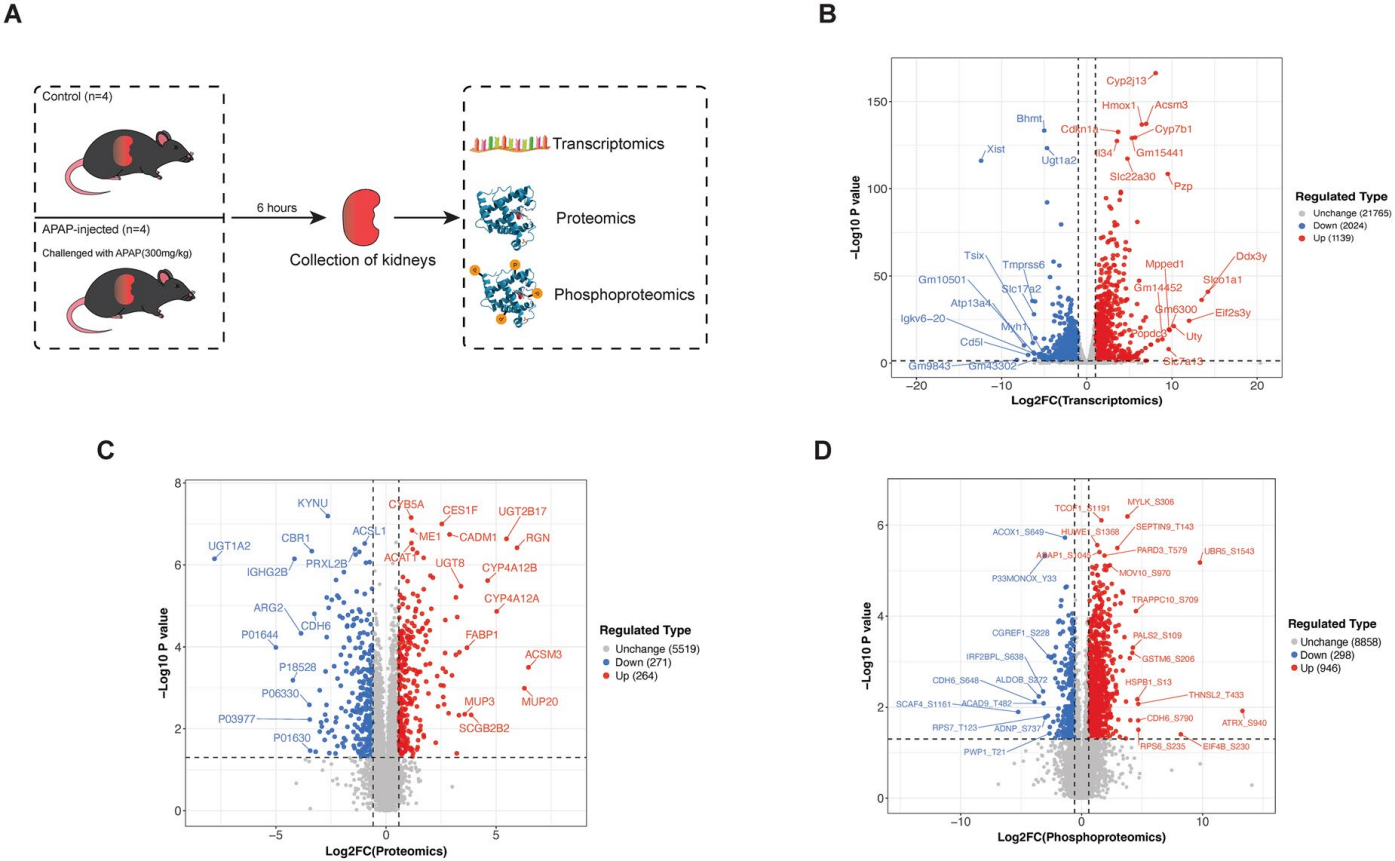
Workflow and global transcriptomic, proteomic, and phosphoproteomic profiling of the kidneys in APAP-induced mice. A. Overview of the study workflow. Eight 10-week-old male C57BL/6 mice were evenly divided into two groups: control (n = 4) and APAP-treated (n = 4). Mice in the APAP group received an intraperitoneal injection of 300 mg/kg APAP, while control mice were given equal volumes of PBS. Six hours post-injection, all mice were euthanized, and their kidneys were collected for transcriptomic, proteomic, and phosphoproteomic analyses. B. Volcano plot showing quantifiable and differentially expressed genes in the transcriptome. Genes with a fold change (FC) > 2 (upregulated) or FC < 1/2 (downregulated) and a Student’s t-test *P_adjust_* value < 0.05 were considered differentially expressed. C. Volcano plot displaying quantifiable and differentially expressed proteins in the proteome, with proteins defined as differentially expressed if FC > 1.5 (upregulated) or FC < 1/1.5 (downregulated), and a Student’s t-test *P* value < 0.05. D. Volcano plot of quantifiable and differentially expressed phosphorylation sites in the phosphoproteome. Phosphorylation sites were deemed differentially expressed based on an FC > 1.5 (upregulated) or FC < 1/1.5 (downregulated), with a Student’s *t*-test *P* value < 0.05.

Eight 10-week-old male C57BL/6 mice were evenly divided into two groups: a control group (*n* = 4) and an APAP-injected group (*n* = 4). Mice in the APAP-injected group received a 300 mg/kg intraperitoneal injection of APAP, while the control group received an equivalent volume of PBS. Six hours after the injection, all mice were euthanized, and their kidneys were harvested for transcriptomic, LC-MS-based proteomic, and phosphoproteomic analyses (Figure 1A).

For the initial bioinformatics analysis, we compared the APAP-injected group with the control group using Student’s t-test for differential analysis. In the transcriptomic analysis, we identified 24,928 quantifiable genes, of which 1,139 were upregulated and 2,024 were downregulated (*P_adjust_* < 0.05, FC > 2 or < 1/2) (Figure 1B). In the proteomic analysis, 6,054 proteins were quantified, with 264 upregulated and 271 downregulated (*p* < 0.05, FC > 1.5 or < 1/1.5) (Figure 1C). The phosphoproteomic analysis identified 10,102 quantifiable phosphosites across 2,706 proteins, with 946 phosphosites upregulated and 298 downregulated (*p* < 0.05, FC > 1.5 or < 1/1.5) (Figure 1D).

### 3.2. Distinct and shared functional enrichments between the transcriptome and proteome in APAP-treated mice

**Figure 2.**
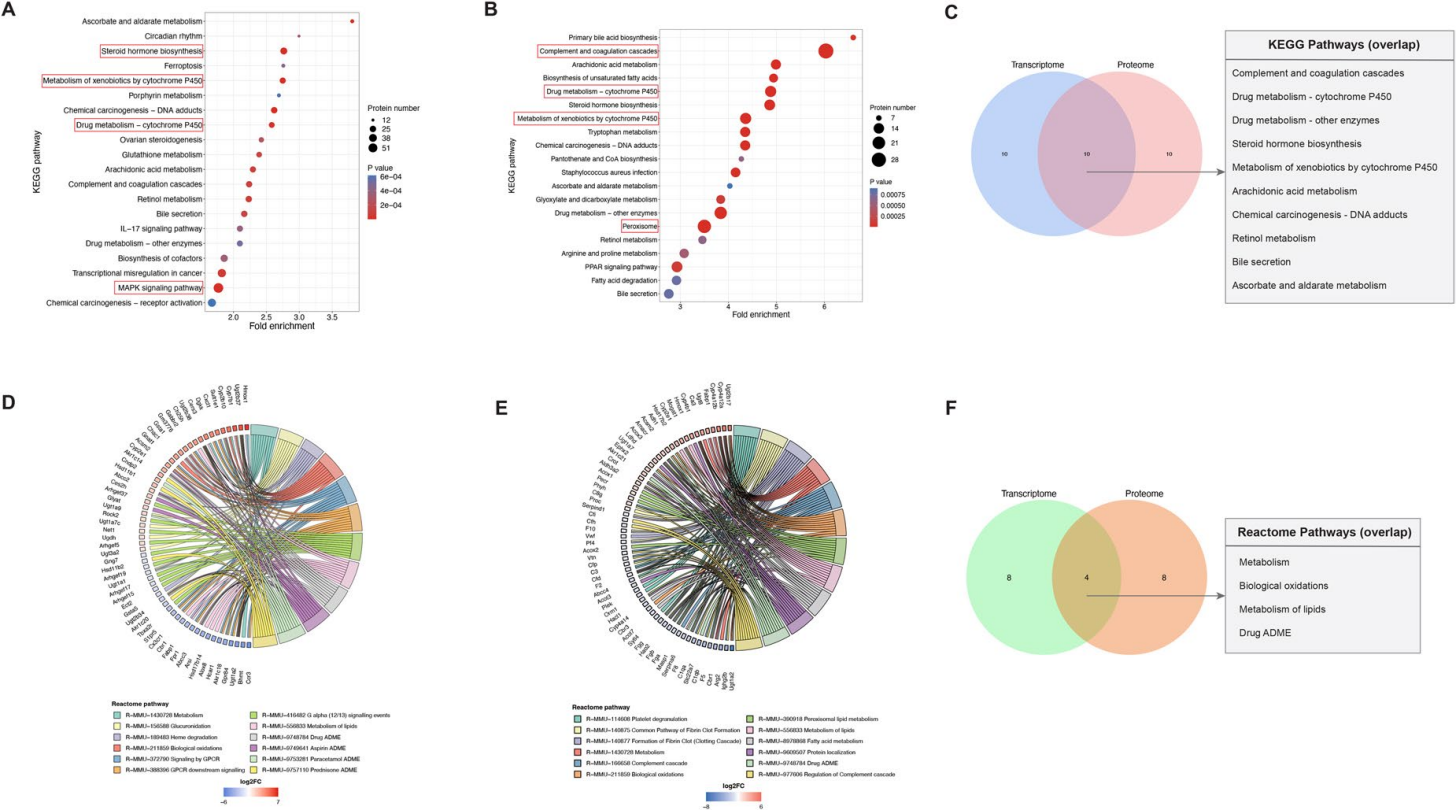
Functional analysis of differentially expressed genes and proteins. A. KEGG pathway enrichment analysis of differentially expressed genes (DEGs) in the transcriptome. DEGs were identified with an FC > 2 (upregulated) or FC < 1/2 (downregulated), and a Student’s *t*-test *P_adjust_* value < 0.05. The top 20 KEGG pathways are presented in bubble plots. B. KEGG pathway enrichment analysis of differentially expressed proteins (DEPs) in the proteome. DEPs were defined as FC > 1.5 (upregulated) or FC < 1/1.5 (downregulated), with a Student’s *t*-test *P* value < 0.05. The top 20 KEGG pathways are shown. C. Overlap of KEGG pathway enrichment between the transcriptome and proteome. D. Reactome pathway enrichment for DEGs in the transcriptome. The top 12 Reactome pathways are illustrated in circos plots. E. Reactome pathway enrichment for DEPs in the proteome. F. Overlap of Reactome pathway enrichment between the transcriptome and proteome.

To identify molecular alterations induced by APAP exposure, we performed differential expression analysis, classifying genes with *P_adjust_* < 0.05 and |fold change| > 2 as differentially expressed genes (DEGs), and proteins with *p* < 0.05 and |fold change| > 1.5 as differentially expressed proteins (DEPs). Subsequent KEGG and Reactome pathway enrichment analyses were conducted to interpret their functional relevance. The top 20 KEGG and top 12 Reactome pathways were visualized using bubble and circos plots, respectively (Figure 2A, B, D, E), enabling a comparative view of unique and overlapping biological pathways in the kidneys of APAP-treated mice.

Transcriptomic profiling revealed prominent enrichment of DEGs in MAPK signaling, drug metabolism – cytochrome P450, metabolism of xenobiotics by cytochrome P450, and steroid hormone biosynthesis (Figure 2A). Correspondingly, Reactome enrichment emphasized biological oxidation and drug metabolism, including pathways specific to paracetamol and aspirin metabolism (Figure 2D), reflecting the transcript-level response to APAP’s biotransformation.

Proteomic data, with subtle distinctions, highlighted changes in complement and coagulation cascades, peroxisome function, drug metabolism – cytochrome P450, and metabolism of xenobiotics by cytochrome P450 (Figure 2B), indicating a stronger emphasis on cytochrome P450 related metabolism and inflammatory response at the protein level. Reactome analysis also supported enrichment in drug metabolism, complement cascade regulation, and biological oxidations (Figure 2E), further suggesting metabolic and immune system perturbations.

Notably, several pathways were convergently enriched in both datasets, including complement and coagulation cascades, drug metabolism, steroid hormone biosynthesis, and arachidonic acid metabolism (Figure 2C, F). These shared pathways reflect the systemic impact of APAP metabolism by cytochrome P450 and validate the robustness of our multi-omics approach in capturing key biological responses at both transcript and protein levels. The divergence observed between the datasets may highlight post-transcriptional regulation, time-dependent dynamics, or protein turnover effects, which warrant further exploration.

### 3.3. Identification of PCD-related genes and pathway activity analysis between control and APAP-induced nephrotoxicity

**Figure 3.**
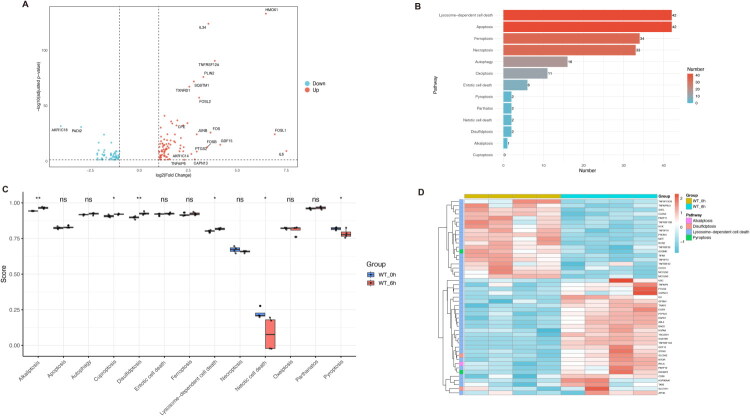
Programmed cell death (PCD)-related gene expression and pathway activity analysis in control and APAP-induced kidney injury. A. Volcano plot of PCD-related DEGs in the transcriptome. DEGs were identified with an FC > 2 (upregulated) or FC < 1/2 (downregulated), and a Student’s *t*-test *P_ajdust_* value < 0.05. B. A total of 183 DEGs were associated with PCD-related pathways. C. GSVA analysis from transcriptomic data revealed differential activities of 13 PCD pathways, as illustrated by box plots comparing control and APAP-induced kidney injury groups. D. Heatmap of DEGs involved in GSVA analysis highlighting significantly altered pathways, including alkaliptosis, disulfidptosis, lysosome-dependent cell death, and pyroptosis.

**Figure 4.**
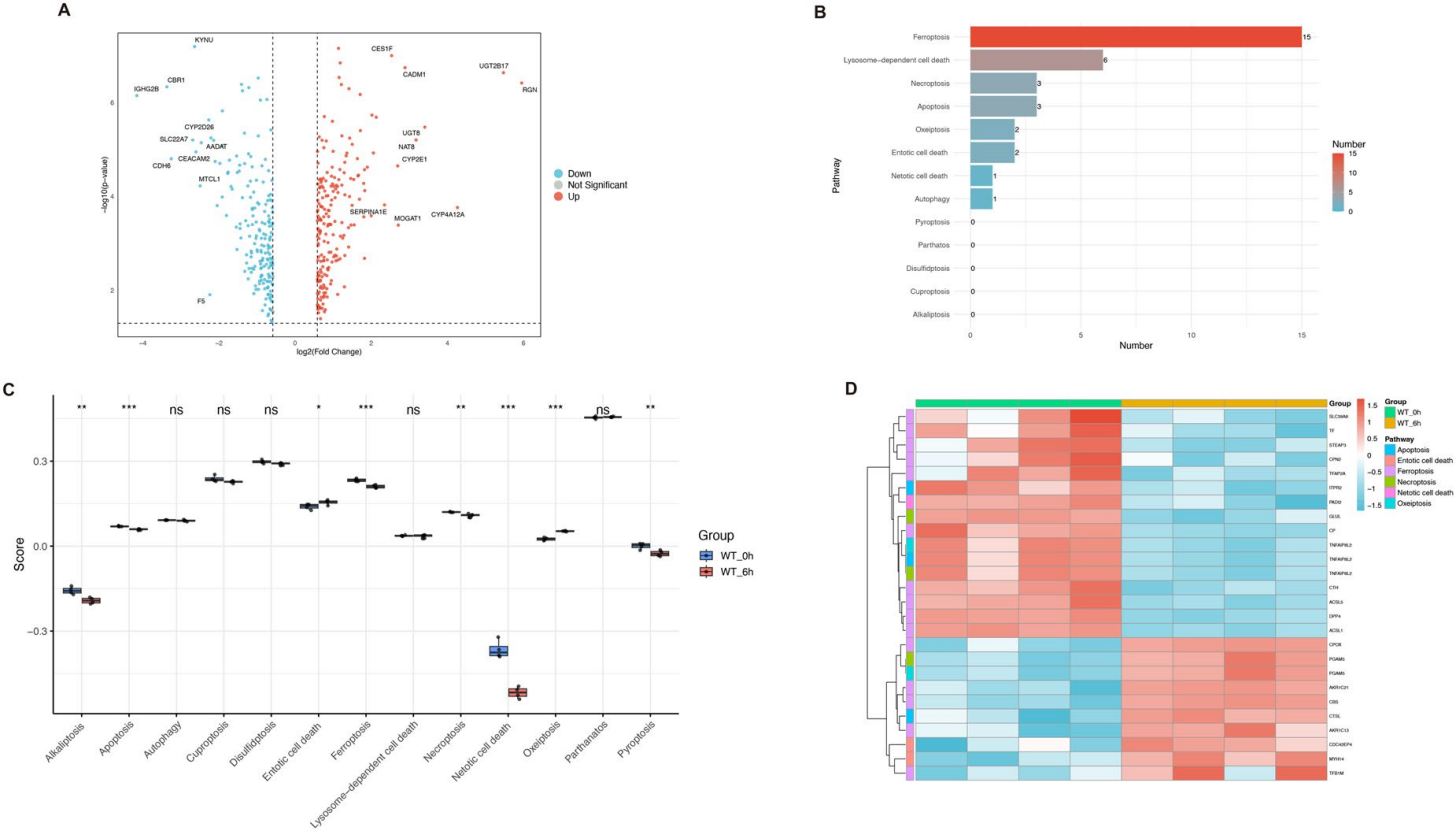
Programmed cell death (PCD)-related protein expression and pathway activity analysis in control and APAP-induced kidney injury. A. Volcano plot of PCD-related DEPs in proteome. DEPs were identified with an FC > 1.5 (upregulated) or FC < 1/1.5 (downregulated), and a Student’s *t*-test *P* value < 0.05. B. A total of 33 DEPs were associated with PCD-related pathways. C. GSVA analysis from proteomic data revealed differential activities of 13 PCD pathways, as illustrated by box plots comparing control and APAP-induced kidney injury groups. D. Heatmap of DEPs involved in GSVA analysis highlighting significantly altered pathways, including apoptosis, entotic cell death, ferroptosis, necroptosis, netotic cell death, and oxeiptosis.

**Figure 5.**
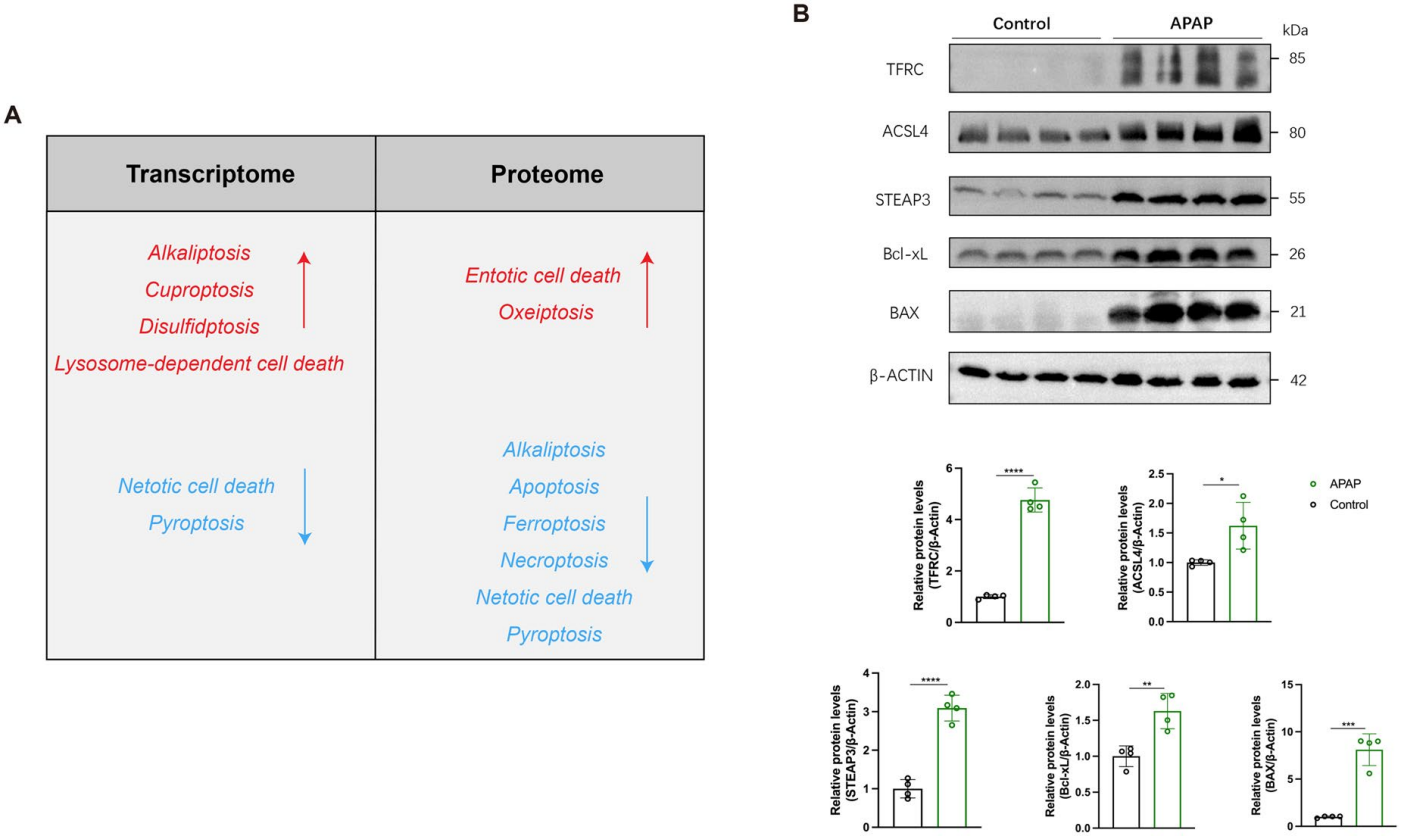
PCD activation in transcriptome vs. proteome, showing representative WB markers for ferroptosis and apoptosis. A. Schematic comparison of PCD activation in the transcriptome and proteome. B. Representative ferroptosis (TFRC, ACSL4, STEAP3) and apoptosis (Bcl-xL, BAX) markers validated by Western blot.

We then analyzed the differences in PCD-related genes between the normal control and nephrotoxicity groups. Our volcano plot and bar chart revealed that among all the cell death-related genes, those within the gene sets for lysosome-dependent cell death, apoptosis, ferroptosis, necroptosis, autophagy, oxeiptosis, entotic cell death, pyroptosis, parthatos, netotic cell death, disulfidptosis, and alkaliptosis exhibited differential expression in the control and APAP-induced nephrotoxicity groups (Figure 3A, B; Figure 4A, B). To further investigate the activation status of various programmed cell death (PCD) pathways in APAP-induced nephrotoxicity, we performed Gene Set Variation Analysis (GSVA) to calculate enrichment scores for 13 PCD-related pathways at both the transcriptomic and proteomic levels. Transcriptomic analysis revealed upregulation of four pathways—alkaliptosis, cuproptosis, disulfidptosis, and lysosome-dependent cell death—in kidneys following APAP-induced injury, whereas netotic cell death and pyroptosis exhibited decreased activity (Figure 3C). In contrast, proteomic analysis showed increased activity in two pathways—entotic cell death and oxeiptosis—while six pathways, namely alkaliptosis, apoptosis, ferroptosis, necroptosis, netotic cell death and pyroptosis, were downregulated (Figure 4C). Differentially expressed genes (DEGs) and differentially expressed proteins (DEPs) within significantly regulated PCD pathways were visualized using heatmaps (Figure 3D and Figure 4D). A schematic diagram comparing PCD activation at the transcriptomic and proteomic levels is presented in Figure 5A. Representative markers of ferroptosis (TFRC, ACSL4, STEAP3) and apoptosis (Bcl-xL, BAX) were further examined (Figure 5B). The expression of TFRC, ACSL4, and STEAP3, all of which promote ferroptosis, was significantly upregulated in the APAP-induced nephrotoxicity group. Similarly, both Bcl-xL (anti-apoptotic) and BAX (pro-apoptotic) were upregulated, although the increase in BAX was more pronounced than that of Bcl-xL. These results indicate potential differences in PCD regulation; however, due to the inherent limitations of proteomic data, the actual activation status of PCD requires further validation. Collectively, these findings suggest that PCD plays a critical role in the development and progression of APAP-induced nephrotoxicity.

### 3.4. Recognition of differential PCD-related genes both in transcriptome and proteome, and construction of protein-protein interaction (PPI) networks

**Figure 6.**
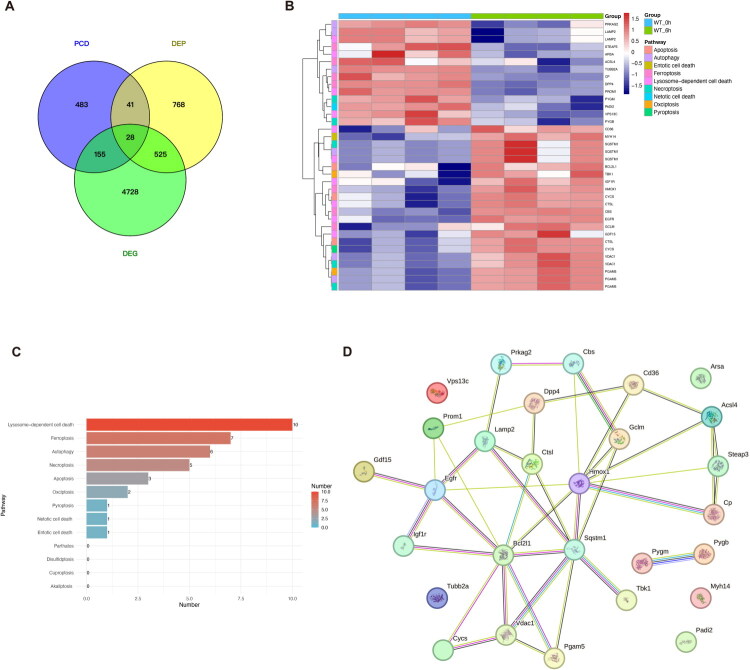
Construction of protein-protein interaction (PPI) networks in the differentially expressed PCD-related genes. A. Identification of PCD-related genes in differentially expressed genes (DEGs) and differentially expressed proteins (DEPs). Totally 28 differentially expressed PCD-related genes were found. A less stringent threshold for differential expression analysis, setting the fold change (FC) cutoff at 1.5 for transcriptomic data and 1.2 for proteomic data. B. Heatmap of 28 overlapping differentially expressed genes, with their associated PCD pathways indicated. C. The bar chart shows the PCD pathways to which the 28 differentially expressed PCD-related genes belong. D. Protein–protein interaction (PPI) networks were constructed using differentially expressed PCD-related genes via the STRING database.

To further explore the central molecular players involved in APAP-induced kidney injury, we adopted a less stringent threshold for differential expression analysis, setting the fold change (FC) cutoff at 1.5 for transcriptomic data and 1.2 for proteomic data. Using these criteria, we identified 28 PCD-related genes that were differentially expressed at both the mRNA and protein levels (Figure 6A–C). To gain insight into their potential interactions and functional relevance, a protein–protein interaction (PPI) network was constructed based on these overlapping genes (Figure 6D). Network topology analysis highlighted five hub genes—Bcl2l1, Hmox1, Sqstm1, Egfr, and Ctsl—which exhibited the highest interaction degrees. These genes are functionally linked to apoptosis regulation (Bcl2l1), oxidative stress responses (Hmox1), autophagy and Nrf2 signaling (Sqstm1), growth factor signaling and repair (Egfr), and lysosomal degradation (Ctsl), suggesting their potential central roles in mediating APAP-induced nephrotoxicity. Moreover, as these processes are broadly implicated in drug-induced kidney injury caused by agents such as cisplatin, antibiotics, and NSAIDs, the identified hub genes may represent not only key mediators of APAP toxicity but also promising candidates for future investigation in the wider context of nephrotoxicity.

### 3.5. Kinase prediction and kinase-substrate regulatory network construction

**Figure 7.**
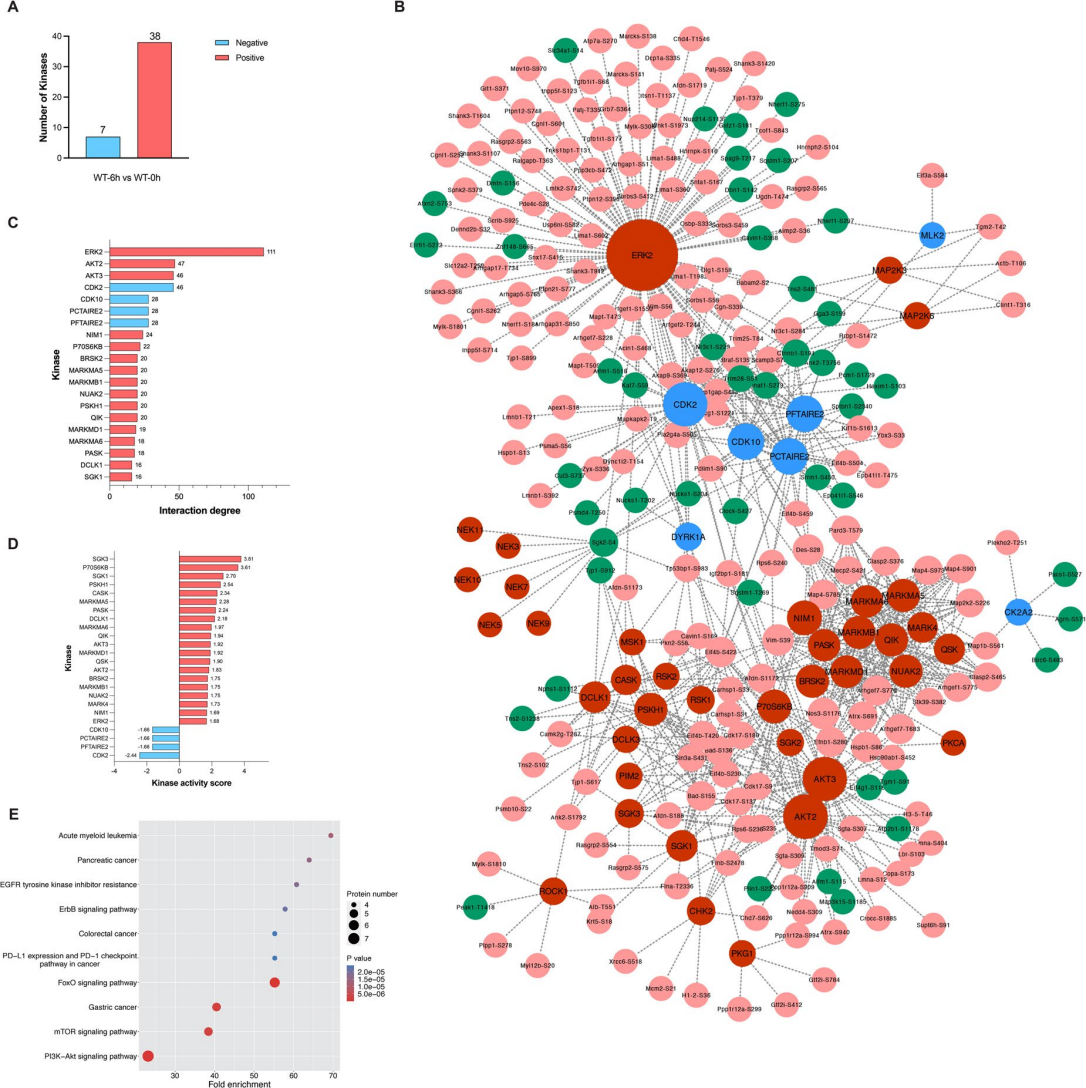
Kinase prediction, activity, and kinase-phosphosite interactions derived from phosphoproteomic analysis. A. Predicted kinase activities following APAP challenge. Kinase prediction was based on phosphorylation site quantification using the iGPS algorithm, and kinase activity prediction was performed using the KSEA algorithm. Normalized enrichment scores (z.score) indicate kinase activity, with positive z.scores for activated kinases and negative z.scores for inhibited kinases. B. Construction of a kinase-substrate regulatory network using differentially expressed phosphosites (FC > 1.5 for upregulated or FC < 1/1.5 for downregulated, Student’s *t*-test *P* value < 0.05). Red nodes represent activated kinases, blue nodes inhibited kinases, pink nodes upregulated phosphosites, and green nodes downregulated phosphosites. Node size reflects interaction degree. C. Top 20 kinase-phosphosite interactions, including both activated and inhibited kinases, are displayed as a bar chart. D. Significant kinase activity (*P* value < 0.05, interaction degree > 10) is presented as a bar chart, based on normalized enrichment scores from the KSEA algorithm. E. KEGG enrichment analysis on kinases with interaction degree of ≥10.

To further dissect the phosphorylation-driven signaling alterations in APAP-induced nephrotoxicity, we performed kinase activity inference using iGPS for kinase-substrate prediction and KSEA (Kinase-Substrate Enrichment Analysis) to estimate kinase activity changes. Based on KSEA-derived z-scores, 38 kinases were predicted to be activated and 7 were inhibited following APAP exposure, indicating widespread modulation of kinase signaling in the kidney (Figure 7A).

We then constructed a kinase-substrate regulatory network using differentially expressed phosphosites. This analysis identified ERK2, AKT2, and AKT3 as central hub kinases with high substrate connectivity, suggesting their pivotal role in coordinating downstream phosphorylation events (Figure 7B). The top 20 kinase–phosphosite interactions were further visualized to highlight the most prominent regulatory relationships (Figure 7C), and key kinase activities were quantitatively assessed (Figure 7D).

To gain insight into the biological pathways governed by these kinases, KEGG enrichment was performed on kinases with a high degree of interaction (≥10). The analysis revealed significant enrichment in FoxO signaling, PI3K-Akt signaling, mTOR signaling, as well as cancer-related pathways such as gastric cancer and acute myeloid leukemia (Figure 7E). These findings highlight the involvement of pro-survival and stress-related signaling cascades in APAP-induced kidney injury, and further support the selection of AKT and ERK as therapeutic targets.

### 3.6. AKT kinase inhibitor alleviated APAP-induced kidney injury

**Figure 8.**
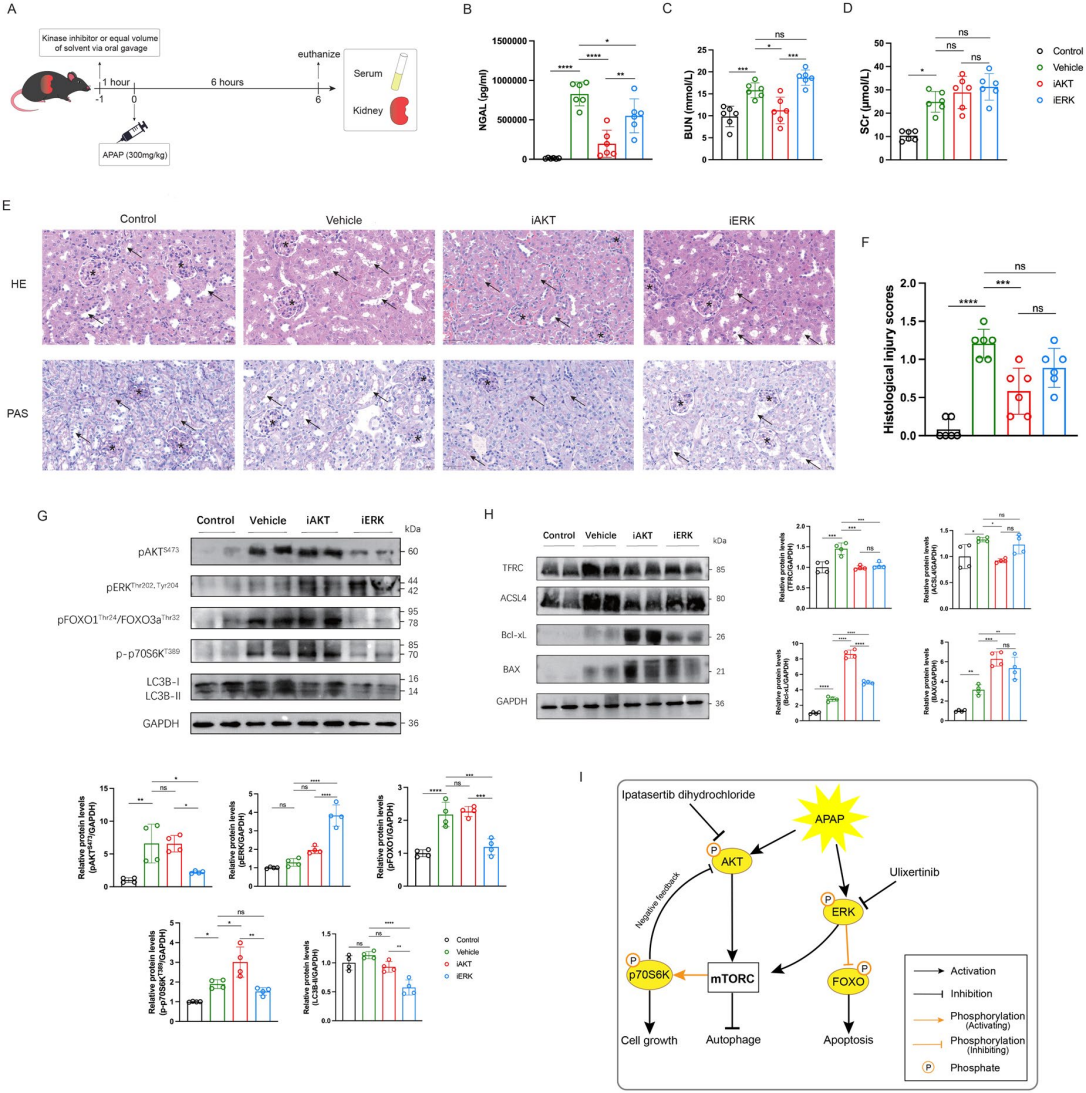
AKT kinase inhibitor reduced APAP-induced kidney injury. A. Overview of the in vivo kinase inhibition experiment. Ten-week-old male C57BL/6 mice were divided into four groups: normal control, vehicle, AKT inhibition (iAKT), and ERK inhibition (iERK) (n = 6). In the kinase inhibition groups, mice were pretreated with specific inhibitors (ipatasertib dihydrochloride or ulixertinib) via oral gavage 1 hour before APAP injection. The vehicle group received the equal solvent orally. All groups, except the control group, received an intraperitoneal injection of APAP (300 mg/kg), and all mice were euthanized 6 hours post-APAP administration. B-D. Serum Measurements: Serum levels of (B) NGAL, (C) BUN, and (D) SCr were measured across groups. E. Kidney sections were stained using HE and PAS. The asterisk indicates the glomerulus, and the arrow points to the proximal tubule. F. Histological injury scores were evaluated using a blinded scoring system. G. Representative images of western blotting and relative protein expression of pAkt (S473), pErk (Thr202, Tyr204), pFoxO1 (Thr24)/FoxO3a (Thr32), p-p70S6K (T389) in different groups. H. Representative images of western blotting and relative protein expression of TFRC, ACSL4, Bcl-xL, BAX in different groups. I. Schematic diagram depicting the regulatory mechanisms of kinase inhibitors in APAP-induced kidney injury. mTORC, mammalian target of rapamycin complex. *p < 0.05, **p < 0.01, ***p < 0.001, and ****p < 0.0001.

Previous studies have shown that AKT and ERK kinases play critical roles in regulating various forms of programmed cell death [38–43]. Building upon our phosphoproteomic analysis and kinase–substrate interaction predictions, we observed significantly elevated activity scores for both AKT and ERK in APAP-induced kidney injury. Given their potential involvement in modulating key PCD pathways identified earlier, AKT and ERK were selected for further *in vivo* inhibition experiments to evaluate their therapeutic relevance. The experimental workflow is outlined in Figure 8A. Serum NGAL levels, an early biomarker of kidney injury, were significantly reduced in both the iAKT and iERK groups compared to the vehicle group (Figure 8B). Additionally, BUN levels were lower in the iAKT group, suggesting milder kidney damage (Figure 8C). Histological analysis further confirmed the protective effects of AKT inhibition, showing a significant restoration of the brush border, reduced cast formation, and decreased desquamation of damage tubular epithelial cells in the iAKT group compared to the vehicle group (Figure 8E, F). To explore the molecular mechanisms underlying AKT and ERK inhibition, we performed Western blot analysis on kidney tissue samples from the Control, Vehicle, iAKT, and iERK groups. Based on pathway enrichment analysis (Table 2), we examined phosphorylation levels of key molecules in the FoxO, PI3K-Akt, and mTOR signaling pathways. Consistent with previous findings [44,45], ATP-competitive inhibitors such as ipatasertib dihydrochloride and ulixertinib bind to the active sites of AKT or ERK, shielding them from phosphatases and thereby increasing pAKT or pERK levels (Figure 8G). Despite this increase, downstream signaling activity was inhibited. As shown in Figure 8G, phosphorylation of the ERK downstream target FoxO was suppressed in the iERK group. Due to the complex feedback regulation involving p70S6K and ERK activation [46,47], AKT inhibition also disrupted negative feedback loops, leading to enhanced phosphorylation of p70S6K, which promotes cell growth. AKT inhibition also reduced the expression of ferroptosis-promoting markers TFRC and ACSL4, while increasing the anti-apoptotic marker Bcl-xL (Figure 8H). Although the iERK group exhibited significantly reduced autophagy, the concurrent decrease in p70S6K phosphorylation (which supports cell growth) and FoxO phosphorylation (which counteracts apoptosis) may explain why the renoprotective effects in this group were less pronounced compared to the iAKT group (Figure 8G). A summary of the regulatory mechanisms of kinase inhibitors is illustrated in Figure 8I.

**Table 2.** Kinases enriched in the top five KEGG pathways.

Pathways	P value	Kinases	Count	Fold Enrichment
FoxO signaling pathway	8.81E-09	ERK2, CDK2, AKT2, SGK1, SGK3, AKT3	6	55.22
PI3K-Akt signaling pathway	1.89E-08	P70S6KB, ERK2, CDK2, AKT2, SGK1, SGK3, AKT3	7	23.17
Gastric cancer	1.84E-06	P70S6KB, ERK2, CDK2, AKT2, AKT3	5	40.49
mTOR signaling pathway	2.27E-06	P70S6KB, ERK2, AKT2, SGK1, AKT3	5	38.44
Acute myeloid leukemia	1.23E-05	P70S6KB, ERK2, AKT2, AKT3	4	69.41

## 4. Discussion

In this study, we performed integrated transcriptomic, proteomic, and phosphoproteomic analyses in a mouse model of APAP-induced kidney injury. We found that multiple programmed cell death pathways are involved, including apoptosis and ferroptosis, highlighting kidney-specific mechanisms that differ from hepatic pathways and therefore require distinct therapeutic considerations. Consistent with KDIGO criteria, elevations in serum creatinine and BUN, together with NGAL as an early tubular injury marker, provided functional evidence of AKI and linked our molecular findings to translational biomarker frameworks. Phosphoproteomic profiling further identified AKT as a central kinase node, while its inhibition alleviated renal injury *in vivo.* These findings should be regarded as hypothesis-generating and warrant validation through additional mechanistic and translational studies.

In this study, we comprehensively evaluated the involvement of 13 PCD modalities in APAP-induced nephrotoxicity through integrated transcriptomic and proteomic analyses. Our findings revealed extensive alterations in both the expression of PCD-related genes and the activity of corresponding pathways, highlighting the complex and multifaceted nature of cell death regulation during APAP-mediated kidney injury. Notably, lysosome-dependent cell death, ferroptosis, necroptosis, and apoptosis gene sets showed significant differential expression between control and APAP-treated groups, suggesting their potential contribution to renal injury progression. GSVA analysis further demonstrated distinct regulatory patterns across transcriptomic and proteomic levels. For instance, lysosome-dependent cell death, alkaliptosis, cuproptosis, and disulfidptosis were transcriptionally activated, whereas pyroptosis and netotic cell death exhibited suppressed activity, indicating possible compensatory or context-specific responses to APAP toxicity. At the proteome level, the downregulation of several classical PCD pathways—such as ferroptosis, necroptosis, and apoptosis—contrasted with the upregulation of oxeiptosis and entotic cell death, implying a shift in dominant cell death mechanisms at different regulatory layers. These discrepancies underscore the necessity of multi-omics approaches in accurately deciphering cell death dynamics. Nevertheless, the inherent limitations of proteomic data mean that the true activation status of PCD remains to be validated. Collectively, our results suggest that APAP-induced kidney injury is not driven by a single form of PCD but rather involves coordinated or competing contributions from multiple death pathways, which may represent novel therapeutic targets to attenuate renal damage.

Although the mechanisms underlying APAP-induced liver injury have been extensively characterized, the molecular pathways involved in APAP-induced kidney injury remain poorly understood. While clinical samples are ideal for translational research, their availability is often limited, and mechanistic investigations are generally more feasible in animal models. Our team has established substantial expertise with APAP-induced toxicity models, having previously conducted a series of studies focused on liver injury [48,49]. In the present study, we employed a moderate APAP overdose (300 mg/kg) to investigate early-stage renal injury. At this dosage, kidney damage was relatively mild compared to the more severe hepatic injury observed under similar conditions.

Consistent with previous reports, our transcriptomic and proteomic analyses both indicated pivotal role of the cytochrome P450–mediated drug metabolism in the context of early APAP-induced kidney injury. Cytochrome P450 enzymes, particularly CYP2E1, are responsible for converting APAP into the highly reactive metabolite N-acetyl-p-benzoquinone imine (NAPQI), which initiates oxidative stress and cellular injury. This mechanism has been extensively characterized in the liver, where CYP2E1 is both expressed in endoplasmic reticulum (ER) and mitochondria of hepatocytes, leading to mitochondrial dysfunction, glutathione depletion, and subsequent hepatocyte necrosis. However, the kidney exhibits important differences in CYP2E1 expression and localization. Studies have shown that in renal proximal tubular cells, CYP2E1 is mainly localized to the ER rather than the mitochondria [23]. As such, the downstream cellular events following NAPQI formation in the kidney may not fully recapitulate the well-established hepatic injury model. For instance, Akakpo et al. reported that ER-localized CYP2E1 activity in renal cells leads to sustained ER stress and activation of procaspase-12, thereby promoting ER stress–mediated apoptosis rather than mitochondrial necrosis [23]. These differences suggest that APAP-induced nephrotoxicity involves distinct molecular mechanisms compared to hepatotoxicity and should not be interpreted as a direct extension of liver injury processes.

Moreover, accumulating evidence suggests that multiple injury mechanisms are triggered downstream of cytochrome P450-mediated metabolism. Hu et al. demonstrated that mitochondrial complex I inhibition by rotenone alleviated APAP-induced renal injury by reducing oxidative stress and inflammation [19]. Shen et al. reported that IL-22 treatment ameliorated kidney injury *via* improving mitochondrial function and suppressing NLRP3 inflammasome activation [20]. Ruan et al. further showed that pretreatment with sika deer antler protein attenuated oxidative stress and apoptosis through activation of Nrf2 and suppression of FoxO1 *via* the PI3K/AKT signaling pathway [50].

These findings underscore the multifactorial nature of APAP-induced nephrotoxicity, involving not only reactive metabolite generation but also ER stress, mitochondrial dysfunction, apoptosis, inflammation, and redox imbalance [19,20,51]. Our integrated multi-omics analysis supports this complexity, revealing changes across multiple signaling pathways and the activation of diverse PCD patterns. Collectively, these results highlight that kidney-specific mechanisms of APAP toxicity differ from hepatic pathways and require distinct therapeutic approaches.

Phosphorylation is one of the most prevalent and extensively studied post-translational modifications in eukaryotic proteins, playing a pivotal role in disease regulation. A single kinase can regulate multiple substrates, while a phosphorylation site may be modulated by multiple kinases, adding to the complexity of these regulatory networks. Given this intricacy, we also investigated kinase regulation in APAP-induced kidney injury. In multiple organ injury models, inhibition of AKT signaling has been associated with enhanced apoptosis, increased oxidative stress, and exacerbated tissue damage [52–55]. Extensive evidence highlights the critical role of the AKT pathway in promoting cell survival across various cell types, including renal cells, and underscores its importance in the context of acute kidney injury. For instance, Yu et al. reported that the TRPM2 channel exerts a protective effect in cisplatin-induced AKI by downregulating the AKT-mTOR signaling pathway and enhancing autophagy [56]. In contrast, Ouyang et al. found that overexpression of circ-ZNF609, which encodes ZNF609-250aa, induces apoptosis and exacerbates AKI by impairing autophagic flux through AKT/mTOR signaling activation. However, inhibiting AKT and mTOR signaling reversed the autophagy flux impairment and apoptosis induced by ZNF609-250aa [57]. Our findings align with these studies, as AKT inhibition alleviated kidney injury, evidenced by histological improvements and reductions in NGAL and BUN levels. Similarly, inhibition of ERK kinase has also been shown to confer renoprotective effects. Livingston et al. demonstrated that blocking the MAPK/ERK pathway mitigates renal fibrosis and improves kidney function by suppressing EGR1 and FGF2 expression in maladaptive tubules following ischemic AKI [58]. Likewise, Jung et al. proposed that NOX1 inhibition exerts protective effects in ischemia-reperfusion injury by attenuating ROS-induced ERK signaling [59]. Consistent with these studies, our findings support the detrimental role of ERK kinase, as indicated by a significant reduction in serum NGAL levels upon ERK inhibition.

Furthermore, our study highlights the differential effects of AKT and ERK inhibition on kidney injury, providing new insights into their distinct regulatory mechanisms. Although both inhibitors effectively reduced early kidney injury markers, the iAKT group exhibited greater histological and biochemical improvements compared to the iERK group. Notably, ERK inhibition primarily resulted in the reduction of NGAL, suggesting a more modest yet detectable protective role. This observation indicates that ERK signaling still contributes to APAP-induced renal injury, albeit to a lesser extent than AKT. Such differential outcomes may stem from the intricate feedback loops within the PI3K-Akt-mTOR and ERK signaling pathways, as well as ferroptosis and related processes. Specifically, AKT inhibition not only suppressed downstream survival signaling but also disrupted negative feedback regulation, leading to compensatory activation of p70S6K, which promote cell survival. Conversely, ERK inhibition reduced phosphorylation of FoxO, a key regulator of apoptosis, as well as p70S6K, which supports cell growth. These findings underscore that dual-kinase dynamics between AKT and ERK jointly influence APAP-induced kidney injury, even though their relative contributions differ.

The clinical applications of protein kinase regulation are rapidly expanding, with the most notable successes achieved in oncology, where aberrant signaling pathways drive disease progression [60]. Beyond cancer, kinase inhibitors have also demonstrated utility in autoimmune disorders, viral infections, and neurodegenerative diseases [61–63]. In this context, our findings that the oncology drugs ipatasertib (AKT inhibitor) and ulixertinib (ERK inhibitor) alleviate APAP-induced renal injury may be viewed as an example of drug repurposing. However, these results should be regarded as hypothesis-generating rather than definitive, as further validation through dose–response studies, extended observation, and complementary nephrotoxic models (e.g., cisplatin, antibiotics, NSAIDs) will be required. Taken together, our study not only highlights kinase signaling as a potential regulatory hub in APAP nephrotoxicity but also suggests broader implications for drug-induced kidney injury, warranting continued mechanistic and translational investigation.

Consistent with KDIGO-defined criteria, our APAP-induced AKI model showed significant elevations in serum creatinine, confirming functional impairment [25]. In addition, BUN, which is frequently used as a complementary indicator in preclinical studies, was also elevated. Importantly, NGAL—a widely recognized translational biomarker of AKI—was markedly increased, highlighting the robustness of our model and strengthening the bridge between experimental findings and clinical detection frameworks [26–28]. This alignment with KDIGO-defined criteria and established AKI biomarkers (serum creatinine, BUN, and NGAL) increases the translational impact of our findings and positions our study within the broader framework of AKI biomarker research.

However, this study has several limitations. First, although animal models are indispensable for elucidating mechanistic insights, they may not fully capture the complexity of human renal physiology; therefore, caution is warranted when extrapolating these findings to clinical contexts. Second, we utilized a moderate overdose of APAP (300 mg/kg) and examined renal injury at a single early time point (6 h post-injection), which may not encompass the complete temporal dynamics of injury progression. Future studies employing higher doses and multiple time points will be critical for characterizing the full course of renal pathology. Third, while we identified several death-associated pathways and core regulatory genes potentially involved in APAP-induced kidney injury, their precise functional roles remain to be verified through targeted mechanistic investigations. Fourth, the analysis was conducted using whole kidney tissue, which limits resolution at the cellular level and may mask alterations in specific renal cell types. Application of advanced technologies such as single-cell or spatial transcriptomics and proteomics would enable more granular exploration of cell-type-specific responses. Finally, although our phosphoproteomic profiling revealed several kinases with elevated activity scores, functional interrogation was constrained by the unavailability of suitable pharmacological inhibitors or activators. Future research should aim to validate these kinase targets using genetic manipulation or chemical probes as they become accessible.

In conclusion, our study provides a comprehensive characterization of programmed cell death (PCD) involved in APAP-induced acute kidney injury by integrating transcriptomic and proteomic analyses. These results not only deepen our understanding of the molecular landscape of APAP nephrotoxicity but also nominates AKT as a central regulatory hub in APAP-induced AKI, which merits further exploration in translational nephrotoxicity research.

## Supplementary Material

R scripts used for GSVA analysis.docx

Supplementary Figure1.docx

Supplementary Table1.xlsx

## Data Availability

The datasets used or analyzed during the current study are available from the corresponding author on reasonable request.
