# Supplementary material for: A multi-omics landscape of programmed cell death in acetaminophen-induced acute kidney injury
Source: Ren Fail. 2025 Nov 17;47(1):2580064. doi: 10.1080/0886022X.2025.2580064 (PMC12624967; doi:10.1080/0886022X.2025.2580064)
Supplement: R scripts used for GSVA analysis.docx [file IRNF_A_2580064_SM5382.docx]

R scripts used for GSVA analysis

# Install once only (if not already installed):

# install.packages("BiocManager")

# BiocManager::install(c("GSVA", "GSEABase", "limma"))

# install.packages(c("ggplot2", "dplyr", "tidyr", "ggpubr", "writexl", "ggbreak"))

library(GSVA)

library(GSEABase)

library(limma)

library(ggplot2)

library(dplyr)

library(tidyr)

library(ggpubr)

library(writexl)

library(ggbreak)

# 1. Load the expression matrix (the first column is gene_name, followed by the sample columns).

expr_df <- read.delim("expression_matrix.tsv", header = TRUE, stringsAsFactors = FALSE)

# 2. Remove duplicates and aggregate by gene name

expr_df_agg <- expr_df %>%

group_by(gene_name) %>%

summarise(across(where(is.numeric), mean), .groups = "drop")

# 3. Convert to an expression matrix

expr_matrix <- as.matrix(expr_df_agg[, -1])

rownames(expr_matrix) <- expr_df_agg$gene_name

# 4. Load the PCD gene sets (GMT format)

gene_sets <- getGmt("PCD_genes.gmt")

# 5. Create ssGSEA parameters and run

param <- ssgseaParam(exprData = expr_matrix, geneSets = gene_sets)

gsva_res <- gsva(param)

# 6. Construct grouping information

sample_names <- colnames(gsva_res)

group <- ifelse(grepl("^WT_0h", sample_names), "WT_0h", "WT_6h")

group <- factor(group)

# 7. Perform statistical analysis using limma

design <- model.matrix(~ group)

fit <- lmFit(gsva_res, design)

fit <- eBayes(fit)

limma_res <- topTable(fit, coef = 2, number = Inf, adjust.method = "none")

limma_res$PCD <- rownames(limma_res)

# 8. Reshape into long format for plotting

df_long <- as.data.frame(t(gsva_res)) %>%

mutate(Sample = rownames(.), Group = group) %>%

pivot_longer(-c(Sample, Group), names_to = "PCD", values_to = "Score") %>%

left_join(limma_res[, c("PCD", "P.Value")], by = "PCD") %>%

mutate(p_label = ifelse(P.Value < 0.001, "***",

ifelse(P.Value < 0.01, "**",

ifelse(P.Value < 0.05, "*", "ns"))))

# 9. Export the results

write_xlsx(df_long, "PCD_ssGSEA_scores_and_pvalues.xlsx")

write_xlsx(as.data.frame(gsva_res), "PCD_ssGSEA_matrix.xlsx")

write_xlsx(limma_res, "PCD_limma_pvalues_raw.xlsx")

# 10. Plot a boxplot

max_score <- max(df_long$Score, na.rm = TRUE) * 1.05

cat("Starting to plot the boxplot...\n")

p <- ggplot(df_long, aes(x = PCD, y = Score, fill = Group)) +

geom_boxplot(position = position_dodge(0.8)) +

geom_jitter(position = position_jitterdodge(jitter.width = 0.2, dodge.width = 0.8), size = 1.2, alpha = 0.7) +

geom_text(data = distinct(df_long, PCD, p_label),

aes(x = PCD, y = max_score, label = p_label),

inherit.aes = FALSE, size = 5) +

theme_minimal(base_size = 14) +

labs(title = "ssGSEA Scores of PCDs by Group",

y = "Score", x = "PCD") +

theme(axis.text.x = element_text(angle = 45, hjust = 1),

axis.line = element_line(color = "black"),

axis.ticks = element_line(color = "black")) +

scale_fill_manual(values = c("WT_0h" = "#619CFF", "WT_6h" = "#F8766D"))

print(p)

cat("Plotting completed\n")
