# Supplementary material for: A multi-omics landscape of programmed cell death in acetaminophen-induced acute kidney injury
Source: Ren Fail. 2025 Nov 17;47(1):2580064. doi: 10.1080/0886022X.2025.2580064 (PMC12624967; doi:10.1080/0886022X.2025.2580064)
Supplement: Supplementary Figure1.docx [file IRNF_A_2580064_SM5381.docx]

**Supplementary Figure**

**Supplementary Fig. 1 | Validation of RNA-Seq data using RT-qPCR.** We validated the expression levels of the top 10 differentially expressed genes identified by RNA-Seq (*P_adjust_* < 0.05, TPM > 50, ranked by *P_adjust_* value) through RT-qPCR analysis. Gene expression was normalized to GAPDH, and error bars represent the standard deviation (SD) of biologically replicated RT-qPCR assays. All experiments were conducted in at least three independent replicates.
